# Wild-type Transthyretin Amyloid Deposition in an Ascending Aortic Aneurysm

**DOI:** 10.1016/j.jaccas.2024.102311

**Published:** 2024-03-28

**Authors:** Abbas Hoteit, Faye Victoria C. Casimero, James R. Stone, Duke Cameron, Eric M. Isselbacher, Reza Seyedsadjadi, Hanna K. Gaggin

**Affiliations:** a Cardiology Division, Massachusetts General Hospital, Boston, Massachusetts, USA; b Department of Pathology, Massachusetts General Hospital, Harvard Medical School, Boston, Massachusetts, USA; c Department of Cardiac Surgery, Massachusetts General Hospital, Boston, Massachusetts, USA; d Thoracic Aortic Center and Heart Center, Massachusetts General Hospital, and Harvard Medical School, Boston, Massachusetts, USA; e Department of Neurology, Massachusetts General Hospital, Harvard Medical School, Boston, Massachusetts, USA; f Harvard Medical School, Boston, Massachusetts, USA

**Keywords:** ascending aortic aneurysm, transthyretin amyloidosis

## Abstract

Amyloid deposition in aortic tissue is associated with increased stiffness. We report a patient with ascending aortic aneurysm and chronic abdominal aortic dissection who had significant wild-type transthyretin amyloid deposition on surgical pathology. The patient did not have cardiac involvement on further workup.

## Graphical abstract

**Figure:**
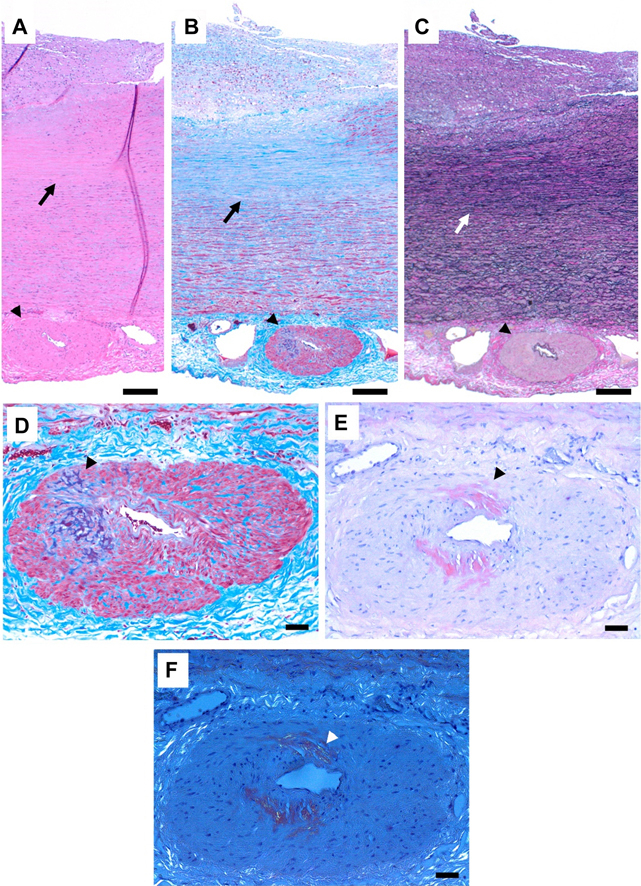

An active, asymptomatic, 64-year-old man with a medical history of hyperlipidemia, pre-diabetes, and spinal stenosis presented for a routine cardiac risk evaluation. He had no significant history of alcohol or tobacco use. His history, physical examination, and electrocardiogram were unremarkable with a normal arm span and joint laxity. Because the patient had an intermediate atherosclerotic cardiovascular disease score of 8.8% and was hesitant about initiating statin therapy, he underwent a coronary calcium scoring computed tomography scan for further evaluation and shared decision-making, which showed, a 4.6-cm ascending aortic aneurysm.

A follow-up echocardiogram showed a tricuspid aortic valve, no evidence of aortic stenosis or regurgitation, a body surface area of 2.2 m^2^, a 4.3 cm aortic root and a 4.4 cm ascending aorta, normal left ventricular cavity size and wall thickness (Supplemental Figure 1). Six-month follow-up imaging with a magnetic resonance angiography of the chest showed an ascending aortic diameter of 4.6 cm (Supplemental Figure 2). In addition, a computed tomography angiogram of the abdomen showed a normal-sized aorta with a chronic dissection originating at the superior mesenteric artery and extending to the left common iliac artery (Supplemental Figure 3).

He had no history of hypertension, family history of thoracic aortic aneurysm, bicuspid aortic valve, or syndromic connective tissue disorders. The most likely etiology of his aortopathy was presumed to be idiopathic congenital medial degeneration. Given the ascending aortic diameter and the fact that he had already dissected a normal caliber abdominal aorta, his ascending aortic aneurysm was considered to be at significantly increased risk for dissection. Consequently, the surgical threshold for his ascending aortic diameter was considered to be 4.5 cm rather than 5.5 cm. He, therefore, underwent open ascending aortic replacement, and the resected ascending aorta was sent for pathological evaluation.

Surgical pathology (Figure 1) showed intimal thickening, medial degeneration (characterized by subintimal smooth muscle cell loss, as highlighted by the trichrome and elastic stains), and amyloid deposits in multiple adventitial vessels of the aorta (as seen on Congo Red staining). No evidence of active aortitis or significant atherosclerosis was seen on pathology. Mass spectrometry on a paraffin block yielded a peptide pattern consistent with transthyretin (TTR) amyloidosis (also known as ATTR). Laboratory evaluation including serum and urine plasma electrophoresis with reflex immunofixation, and serum free light chains was unremarkable (Supplemental Table 1). TTR genetic testing was negative for known pathological mutations. ^99^mTc-pyrophosphate scintigraphy and cardiac magnetic resonance imaging were not suggestive of cardiac amyloidosis (normal biventricular systolic function without late gadolinium enhancements, normal parametric mapping values, normal end-diastolic left ventricular myocardial wall thickness with a normal extracellular volume of 25%). Noninvasive cardiac pulmonary exercise testing with imaging showed preserved exercise capacity with a largely normal central cardiac response to exercise.

**Figure 1:**
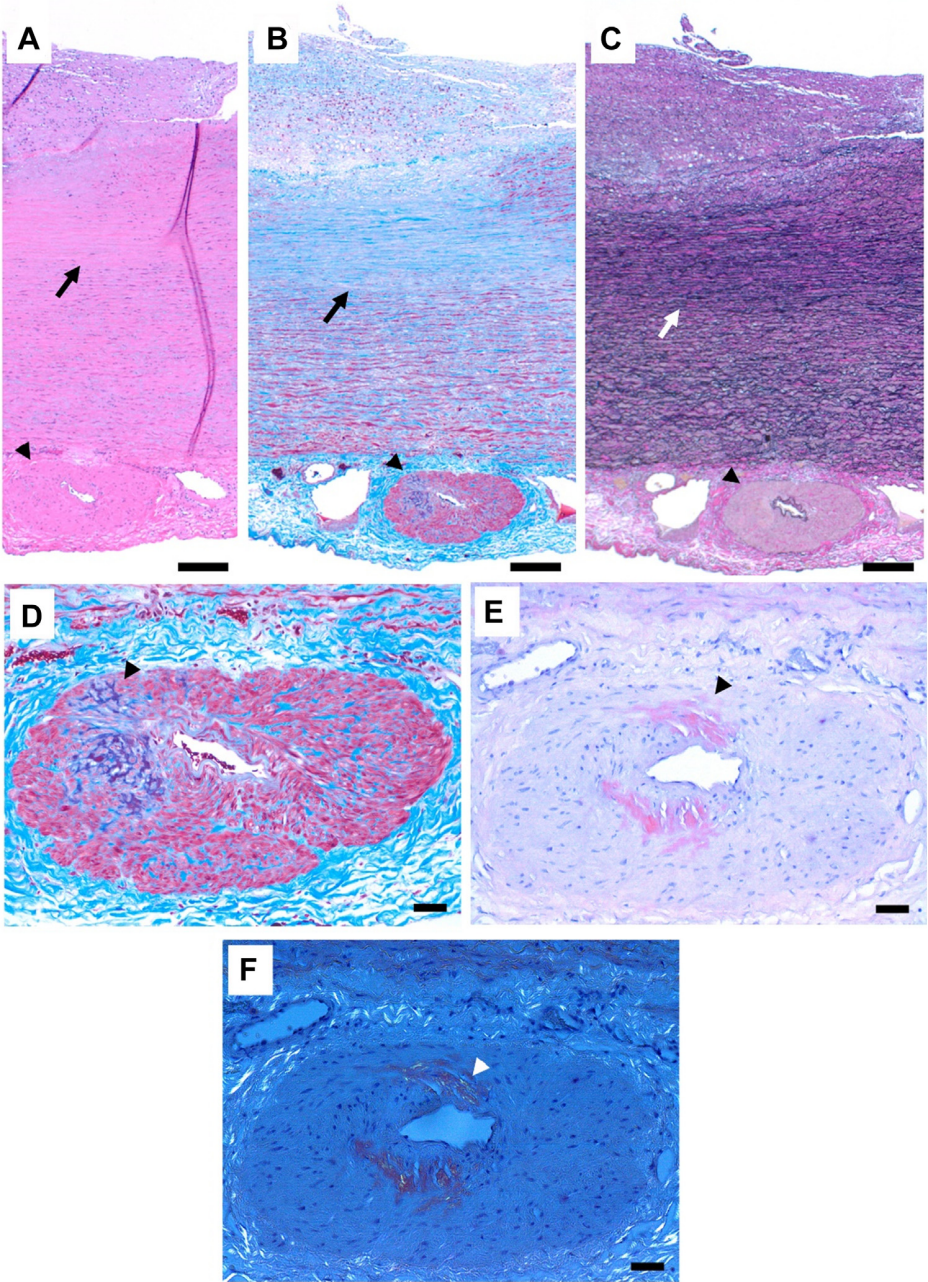
Histopathological Features of the Ascending Aortic Aneurysm with Amyloid Deposition (A to C) Low magnification of the full thickness of the aortic wall stained with hematoxylin and eosin (H&E), trichrome, and elastic stains, respectively. The intima shows thickening and scattered foam cells (top); the media shows subintimal smooth muscle cell loss with laminar collapse (arrows), and the adventitia shows arterial thickening (arrow heads). (D) Trichrome stain highlighting the heterogenous staining deposits in the adventitial artery interspersed between the smooth muscle cells (arrowhead), in contrast with the surrounding blue collagen fibers. (E) Congo red stain showing positive red-orange staining of the amyloid deposits (arrowhead). (F) Congo red stain with application of polarized light showing apple-green birefringence of the amyloid deposits (arrowhead). Scale bars are 200 microns (A to C) and 40 microns (D to F).

Because the workup for TTR amyloid cardiomyopathy was negative, he did not meet the current criteria for treatment with tafamidis, a TTR stabilizer.^1^ After weighing the potential risks and benefits, off-label treatment with diflunisal to slow down the progression of TTR amyloidosis was considered, but ultimately deferred. Although he will be monitored closely for meeting indications for approved disease-modifying therapy for TTR amyloidosis, there are no clear data on how and how often to monitor patients with TTR amyloidosis outside of the heart and the nervous system or how to treat it. As the awareness of TTR amyloidosis grows, we are more likely to find TTR amyloid deposition outside of the heart and the nervous system and the need to address this gap in literature will escalate.

## Funding Support and Author Disclosures

Dr Gaggin has received research grant support from Roche Diagnostics, Pfizer, Alnylam, Akcea (IONIS), and Eidos/BridgeBio; consulting income from Amgen, AstraZeneca, Bayer, Eidos/BridgeBio, Merck, Novo Nordisk, Pfizer, and ExpertConnect; stock option for Eko; research payments for clinical endpoint committees from Baim Institute for Clinical Research for Abbott, Siemens, Innolife, and Beckman Coulter, and from ACI Clinical for Abbott Laboratories; in kind support from the HeartShare fellowship. Dr Hoteit is in part funded by Pfizer’s Transthyretin Cardiac Amyloidosis Fellowship. Dr Stone has received consulting fees from Cook Medical. All other authors have reported that they have no relationships relevant to the contents of this paper to disclose.
