# Supplementary material for: Wild-type Transthyretin Amyloid Deposition in an Ascending Aortic Aneurysm
Source: JACC Case Rep. 2024 Mar 28;29(9):102311. doi: 10.1016/j.jaccas.2024.102311 (PMC10990737; doi:10.1016/j.jaccas.2024.102311)
Supplement: Supplemental Figures 1-3 and Supplemental Table 1 [file mmc1.docx]

| Supplemental Table 1: Laboratory Workup | | |
| --- | --- | --- |
| Laboratory | Value | Reference Range |
| Hemoglobin | 14.3 g/dL | (13.5 – 17.5 g/dL) |
| Platelets | 265 K/µL | (150 – 400 K/µL) |
| Creatinine | 1.03 mg/dL | (0.60 – 1.50 mg/dL) |
| Estimated Glomerular Filtration Rate: | 81 mL/min/1.73m2 | (>59 mL/min/1.73m2) |
| Prealbumin/transthyretin | 25 mg/dL | (20 – 40 mg/dL) |
| Thyroid-Stimulating Hormone | 2.73 µIU/mL | (0.40 – 5.00 µIU/mL) |
| Vitamin A | 54.4 mcg/dL | (32.5 – 78.0 mcg/dL) |
| High sensitivity Troponin T | 22 ng/L | (0 – 14 ng/L) |
| N-terminal B-type natriuretic peptide | 236 pg/mL | (0 – 900 pg/mL) for acute dyspnea for age 50-75 years  (0-125 pg/mL) for ruling out heart failure as outpatient |
| Erythrocyte Sedimentation Rate | 7 mm/h | (0 – 19 mm/h) |
| C-Reactive Protein | 1.1 mg/L | (<8.0 mg/L) |

Supplemental Figure 1: Echocardiogram images in the parasternal long-axis view showing a 4.3 cm aortic root (panel A) and a 4.4 cm ascending thoracic aorta (panel B), as measured using the inner-edge to inner-edge method.

Supplemental Figure 2: Magnetic resonance angiography in the axial plan (panel A) and sagittal plane (panel B) showing a 4.5 x 4.6 cm ascending thoracic aorta.

Supplemental Figure 3: CT angiogram showing a 1.8 cm distal infrarenal abdominal aorta with a localized dissection.
